# SGLT2 inhibition improves sarcomere contractile dysfunction in human models of dilated cardiomyopathy

**DOI:** 10.1186/s12967-026-08702-0

**Published:** 2026-07-31

**Authors:** Daria Plota, Hafiza Nosheen Saleem, Kun-Han Lin, Ruheen Wali, Cleophas Cheruiyot, Luca Münzel, Tabea Hutschenreiter, Malte Tiburcy, Tim Meyer, Michael Habeck, Felix Wiedmann, Constanze Schmidt, Wolfram-Hubertus Zimmermann, Antje Ebert

**Affiliations:** 1 Department of Cardiology and Pneumology, Heart Research Center Goettingen, University Medical Center Goettingen, Georg-August University of Goettingen, Goettingen, Germany; 2 DZHK (German Center for Cardiovascular Research), Partner Site Lower Saxony, Göttingen, Germany; 3 Laboratory of Membrane Biophysics, Max Planck Institute for Multidisciplinary Sciences, Göttingen, Germany; 4 Institute of Pharmacology and Toxicology, University Medical Center Göttingen, Georg August University, Göttingen, Germany; 5 Microscopic Image Analysis Group, University Medical Center Jena, Jena, Germany; 6 Cluster of Excellence ’Multiscale Bioimaging: from Molecular Machines to Networks of Excitable Cells’ (MBExC), University of Göttingen, Göttingen, Germany; 7 Department of Cardiology and Pneumology, Goettingen University Medical Center, Goettingen University, Robert-Koch- Strasse 40, 37075 Goettingen, Germany

**Keywords:** Sarcomere signaling, Mitochondrial iron deficiency, Contractility, CME-dependent signaling, Human iPSC-derived cardiomyocytes, Dilated cardiomyopathy, Signal transduction

## Abstract

**Background:**

Sodium–glucose cotransporter 2 inhibitors (SGLT2i) are now widely applied in treatment plans for heart failure with reduced ejection fraction (HFrEF) patients, regardless of the presence of diabetes. SGLT2i, such as empagliflozin (EMP), reduce the risk of cardiovascular death and heart failure hospitalizations in HFrEF patients. However, the underlying molecular mechanisms of action, by which SGLT2i benefit cardiomyocytes (CMs) in HFrEF hearts, are not well understood.

**Methods:**

This study investigated the role of the SGLT2i, EMP, in a patient-specific human model of dilated cardiomyopathy (DCM), a primary cause of HFrEF, which is frequently caused by inherited mutations in sarcomere proteins. We employed DCM patient-specific induced pluripotent stem cell-derived CMs (iPSC-CMs) carrying the inherited sarcomere protein mutation tropomyosin (TPM1)-L185F together with high-speed motion traction and optical action potential mapping analysis, as well as biochemical methods to dissect molecular signaling dysfunctions.

**Results:**

Our findings indicate that in DCM patient-specific iPSC-CMs, SGLT2i may act via different mechanisms. This includes the regulation of signaling pathways, to modulate sarcomere function via improving mitochondrial respiration and ATP production. As our results show, the SGLT2i, EMP, ameliorates primary sarcomere dysfunctions in DCM TPM1-L185F iPSC-CMs, such as disrupted sarcomere organization, reduced contractile function, and prolonged action potential duration. Moreover, we found that a subcellular signaling pathway dysregulated in DCM CMs, clathrin-mediated endocytosis (CME)-dependent signaling, may be recovered by EMP treatment in DCM TPM1-L185F iPSC-CMs. This pathway is essential for the uptake and distribution of critical cargo, such as transferrin-bound Fe, in CMs. In the presence of the DCM-causing mutation, EMP recovered mitochondrial Fe levels and mitochondrial functional output, which are reduced in DCM iPSC-CMs as part of pathologically defective CME-dependent signaling. These findings support that EMP modulates aspects of CME-signaling, and contributes to restored sarcomere functions. To further elucidate the effects of SGLT2i in DCM CMs, we investigated the molecular functions of a state-of-the-art combinatorial therapy for DCM (HFrEF) patients, the drug combination referred to as “fantastic four” (F4), including an SGLT2i, in DCM iPSC-CMs.

**Conclusions:**

Our in-vitro results point to a significant benefit of F4 to be attributed to the SGLT2i, EMP, regarding sarcomere functions as well as aspects of CME-dependent recovery of molecular signaling. Moreover, studying the recovery of subcellular dysfunctions by EMP treatment, findings from an in-vivo porcine model of HF support improvements observed in the presence of EMP regarding contractile and electrophysiological parameters. Together, our findings provide molecular insights into the subcellular function of SGLT2i and their role as part of the F4 state-of-the-art combination therapy for HFrEF, via targeting different molecular signaling pathways in a human patient-specific 2D model of HFrEF.

**Supplementary Information:**

The online version contains supplementary material available at 10.1186/s12967-026-08702-0.

## Introduction

Sodium–glucose cotransporter 2 inhibitors (SGLT2i) represent a novel drug class with antidiabetic function that were found in multiple clinical trials to substantially improve outcomes of subgroups of patients with forms of HF [1–4]. These advantageous effects of SGLT2i could not be allocated to their glucose-lowering function or modulation of the natriuretic system alone, but may be due to direct mechanistic cardioprotective action of SGLT2i [3, 5, 6]. SGLT2 inhibitors including empagliflozin (EMP) have been approved for clinical management of patients with heart failure with reduced ejection fraction (HFrEF), as well as heart failure with preserved ejection fraction (HFpEF). Left ventricular (LV) end-diastolic and end-systolic volumes are reduced with SGLT2i therapy, indicating a decrease in ventricular dilation [5]. Hemodynamically, SGLT2i lower LV preload, reduce pulmonary artery pressure, and increase cardiac output, which assists to manage congestion and improve cardiac forward flow [7].

Despite their beneficial effects in HFrEF patients, the underlying subcellular and molecular functions of SGLT2 inhibitors in DCM cardiomyocytes (CMs) are to date not fully clear. Advantageous effects of EMP in cardiovascular diseases include improvement of cardiac function, as well as mitochondrial OXPHOS in murine models [8]. At the subcellular level, human iPSC-CM models were employed to demonstrate EMP-based improvement of contractility and electrophysiological properties [9], including reduced arrhythmogenic events via modulation of the action potential duration [10]. However, despite these substantial insights, it is not yet well understood how these favorable effects are mediated by EMP and other SGLT2 inhibitors in CMs. Some research revealed EMP-dependent signaling via modulation of post-translational modifications (PTMs) of critical cardiac proteins, which may directly alter sarcomere function. An example is the increased phosphorylation of phospholamban, the regulator of the sarcoplasmic reticulum Ca^2+^ pump (SERCA), following EMP treatment [10]. This suggests that SGLT2 inhibitors may act directly or indirectly on different signaling pathways, regulating sarcomere functions.

Here, we addressed these points with a human induced pluripotent stem cell (iPSC)-based model of DCM. Human iPSC-derived cardiomyocytes (iPSC-CMs) offer reproducible access to large quantities of developmentally early-stage but functional human cardiomyocytes for molecular patho-mechanistic studies [11, 12]. Previously, human iPSC-CMs were successfully utilized to probe basic molecular dysfunctions in cardiac diseases including familial dilated cardiomyopathy (DCM) [13–18]. Patient-specific iPSC-CMs carrying the familial DCM mutation TPM1-L185F were described earlier [19, 20] and exhibit disrupted sarcomere organization, impaired contractility, reduced force generation, and defective mitochondrial functions [19]. Using this model, we found in patient-specific DCM TPM1-L185F iPSC-CMs, treated with EMP, an improvement of sarcomere organization and contractile function, compared to control vehicle. We next explored the EMP-dependent response of another subcellular signaling mechanism in DCM CMs, clathrin-mediated endocytosis (CME)-dependent signaling. This sub-cellular mechanism modulates sarcomere contractility and organization via mitochondrial Fe levels and mitochondrial function [19] and was reported to be defective in DCM CMs [19, 20]. Reduced mitochondrial Fe levels and mitochondrial function, reflecting defective CME-dependent signaling in DCM CMs [19], were significantly improved following EMP treatment in DCM TPM1-L185F iPSC-CMs, compared to control vehicle.

Of note, in patients with heart failure and reduced ejection fraction (HFrEF), EMP is not a stand-alone therapeutic choice. Instead, patients with HFrEF typically receive a combination therapy of four medications, such as eplerenone (a mineralocorticoid receptor antagonist, MRA); sacubitril/valsartan (an angiotensin-receptor-neprilysin-inhibitor, ARNI) containing the neprilysin inhibitor, sacubitril, and the angiotensin receptor blocker (ARB), valsartan; and metoprolol (a beta-blocker); together with an SGLT2 inhibitor, such as EMP, which has been coined “fantastic four” (F4) [21]. We revealed beneficial consequences of the in-vitro mimicked combination therapy, F4 including EMP, on the sarcomere function of DCM patient-specific iPSC-CMs. Further supporting the positive effect of EMP treatment on cardiomyopathy, a porcine model of arrhythmia-induced cardiomyopathy (AiCM) showed improved cardiac function with a partially preserved left ventricular ejection fraction (LVEF) in the EMP treated group, compared to control animals.

Together, our findings show that the pharmacological SGLT2i, EMP, improves molecular patho-phenotypes in patient-specific iPSC-CMs such as the reduced sarcomere function. Thus, our findings provide deeper insight into the molecular basis of benefits for DCM CMs following treatment with an SGLT2i, EMP, in a human pre-clinical 2D model.

## Results

### DCM patient-specific iPSC-CMs show dysfunctional contractility and action potential (AP) properties

**Fig. 1:**
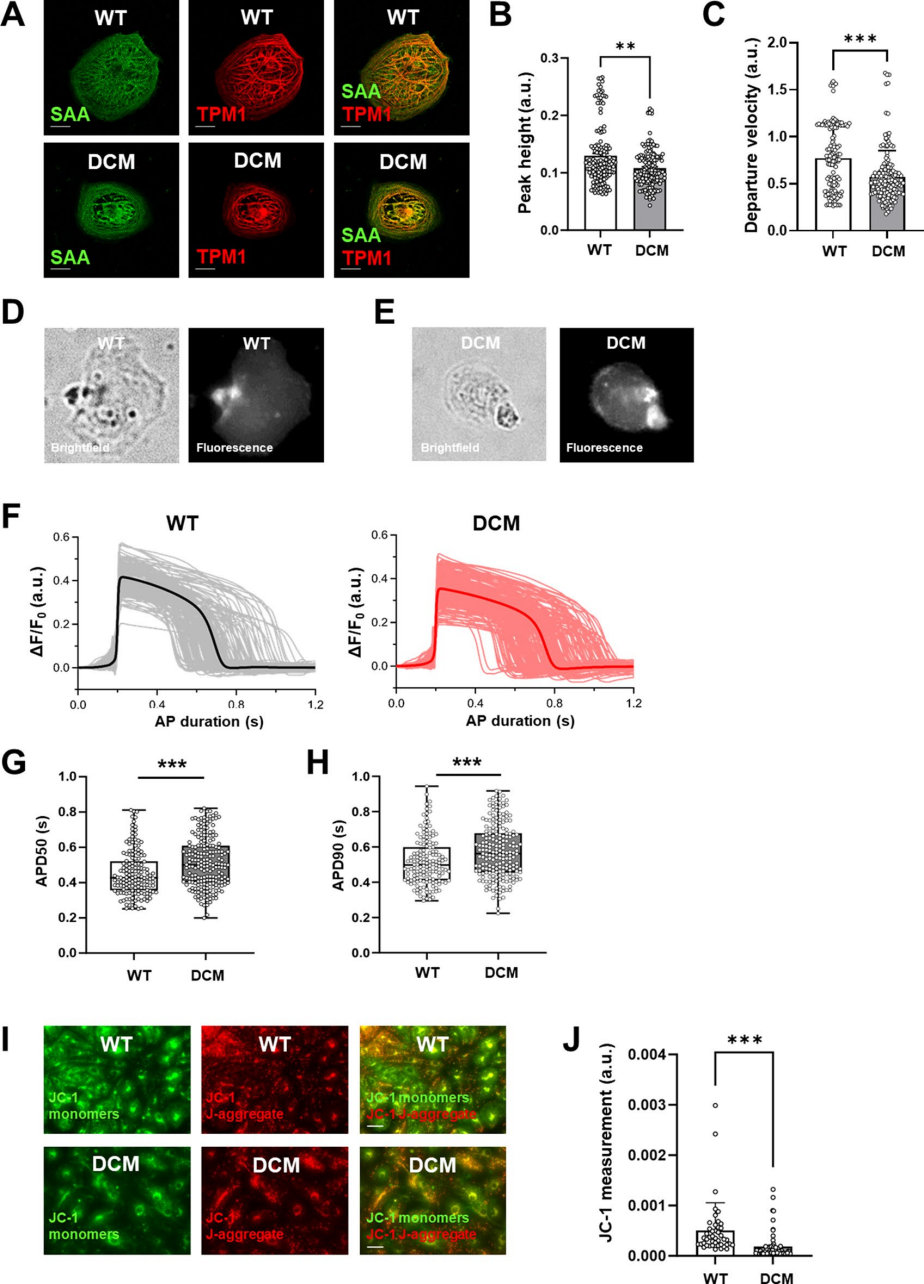
Molecular dysfunctions in DCM patient-specific iPSC-CMs. **A**, Representative 63x confocal images of DCM patient-specific (TPM1-L185F) iPSC-CMs versus WT controls are shown after immunostaining for cardiac troponin T (TnT) (red) and sarcomeric α-actinin (SAA) (green); scale bar, 20 μm. **B**-**C**, Analysis of contractility in monolayers of WT iPSC-CMs (*n* = 159) versus DCM iPSC-CMs (*n* = 145) via the IonOptix motion-traction system; per group 2 independent cardiac differentiations, per experiment 10 recordings. Data are shown as mean ± SEM; a.u., arbitrary units. **B**, Peak height (amplitude), **P < 0.005 (*P* = 0.0016; Mann-Whitney U test). **C**, Departure velocity (speed to peak), ***P < 0.0001 (*P* < 0.0001; Mann-Whitney U test). **D**-**H**, Optical mapping of DCM iPSC-CMs (*n* = 205) versus WT controls (*n* = 153). **D**-**E**, Representative images of recorded single-cell WT (**D**) and DCM (**E**) iPSC-CMs loaded with the voltage-sensitive dye, FluoVolt, in brightfield mode (left) and fluorescence mode (right). **F**, Action potential (AP) duration for WT (left) and DCM (right) iPSC-CMs; **G**, AP duration (APD) 50, ***P < 0.0001 (*P* < 0.0001; Mann-Whitney test); **H**, AP duration (APD) 90, ***P < 0.0001 (*P* < 0.0001; Mann-Whitney test). **I-J**, Mitochondrial membrane potential (JC-1) of DCM iPSC-CMs (*n* = 2 experiments, 44 data points) versus WT controls (*n* = 1 experiment, 44 data points); data are shown as mean ± SEM; a.u., arbitrary units.** I**, Representative 20x images of DCM iPSC-CMs versus WT controls; live cell imaging following staining with the JC-1 dye, indicating monomers (green) and J-aggregates (red); 20x objective; scale bar, 50 μm; **J**, JC-1 measurement (a.u.) ***P < 0.0001 (*P* < 0.0001; Mann-Whitney U test)

**Fig. 2:**
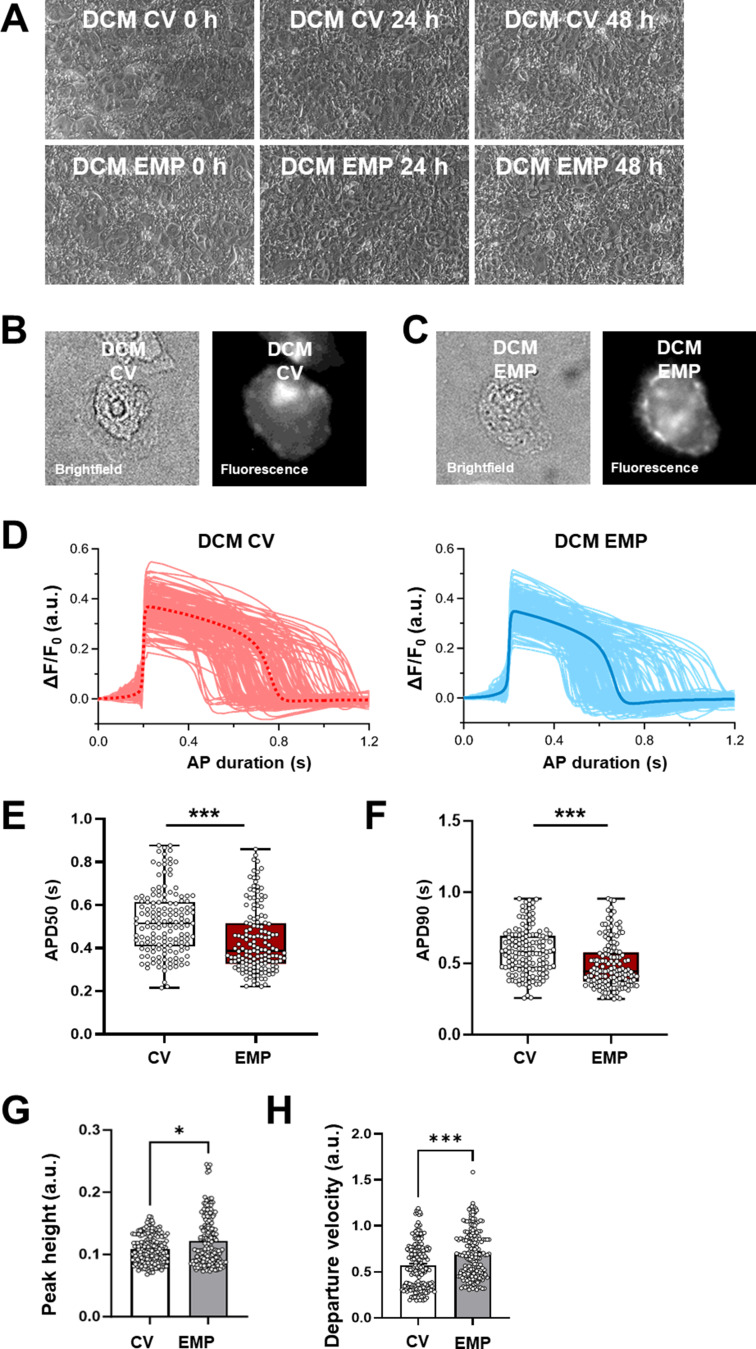
EMP treatment improves contractility and action potential defects in DCM patient-specific iPSC-CMs toward the levels of WT controls. **A**, Representative 20x brightfield images of DCM patient-specific (TPM1-L185F) iPSC-CMs treated with EMP compared to control vehicle at 0 h, 24 h, or 48 h of treatment. **B-F**, Optical mapping of DCM iPSC-CMs treated with EMP (*n* = 148) or control vehicle (*n* = 150). **B-C**, Representative images of recorded single-cell DCM iPSC-CMs treated with control vehicle (**B)** or EMP (**C**) and loaded with the voltage-sensitive dye, FluoVolt, in brightfield mode (left) and fluorescence mode (right). **D**, Action potential (AP) duration for DCM patient-specific iPSC-CMs treated with control vehicle (left) or EMP (right); **E**, AP duration (APD) 50, ***P < 0.0001 (*P* < 0.0001; Mann-Whitney test). **F**, AP duration (APD) 90, ***P < 0.0001 (*P* < 0.0001; Mann-Whitney test). Data are shown as mean ± SEM; a.u., arbitrary units. **G-H**, Contractility recordings via the IonOptix motion-traction platform for DCM iPSC-CMs treated with EMP (*n* = 367 transients) compared to control vehicle (*n* = 346 transients), per group *n* = 3 independent experiments, *n* = 10 recordings per experiment. Data are shown as mean ± SEM; a.u., arbitrary units. **G**, Peak height (amplitude), *P < 0.05 (*P* = 0.0179; Mann-Whitney test) and **H**, Departure velocity (speed to peak), ***P < 0.0001 (*P* < 0.0001, Mann-Whitney test)

**Fig. 3:**
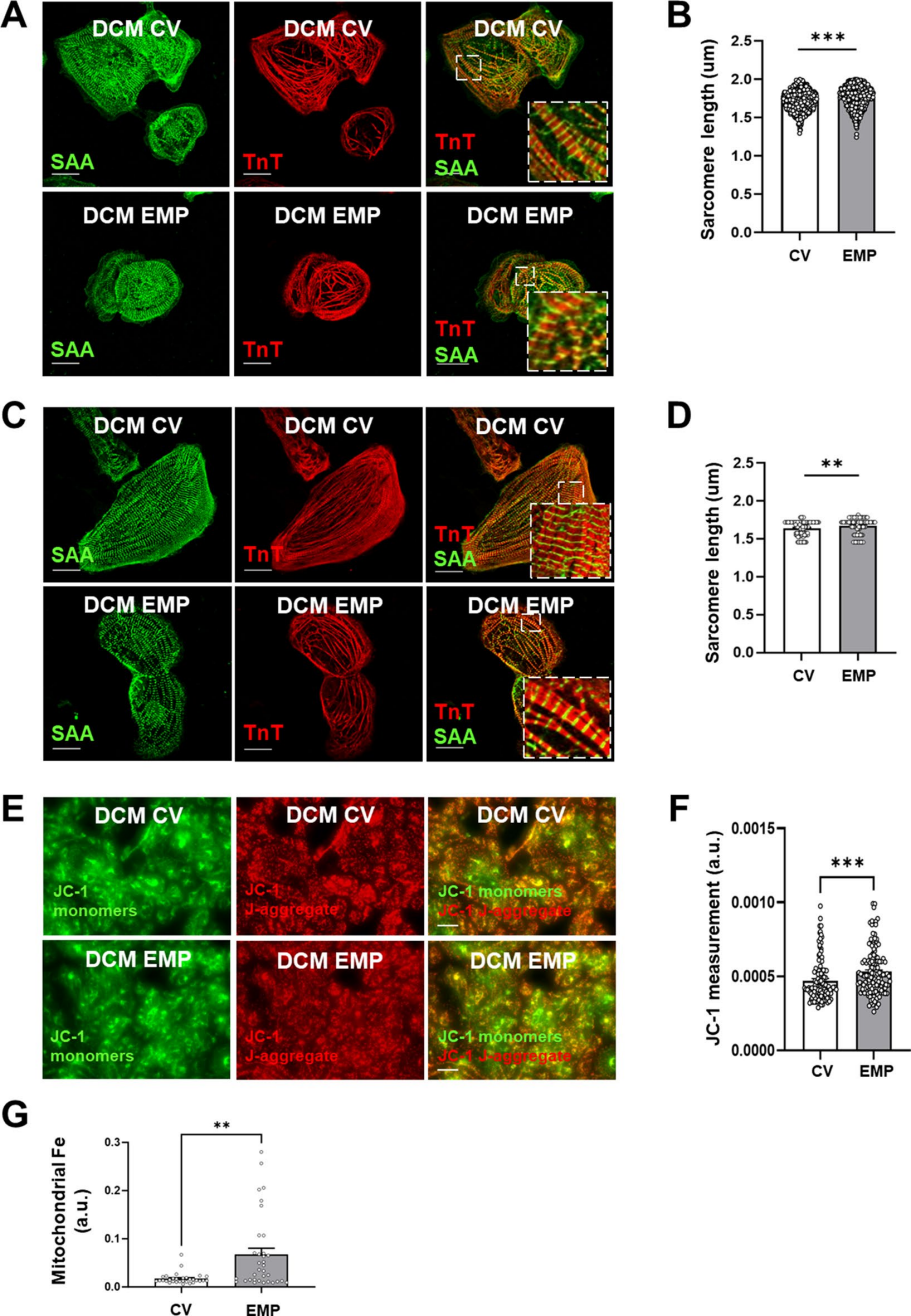
EMP treatment recovers the sarcomere length of DCM patient-specific iPSC-CMs. **A-B**, DCM patient-specific (TPM1-L185F) iPSC-CMs were treated with EMP or control vehicle for 48 h, followed by immunostaining for cardiac troponin T (TnT, red) and sarcomeric α-actinin (SAA, green), as well as confocal imaging. **A**, Representative 63x confocal images are shown; scale bar, 20 μm. **B**, Quantification of the sarcomere length using images described in **A** via our previously reported approach (*15*,* 16*,* 19*); per group, *n* = 3 experiments, *n* = 30 images, *n* = 278 ROIs for EMP-treated DCM iPSC-CMs (1.729 ± 0.005403 μm), *n* = 275 ROIs for CV-treated DCM iPSC-CMs (1.696 ± 0.005595 μm). Data are shown as mean ± SEM; ***P < 0.0001 via Mann-Whitney U test. **C-D**, DCM patient-specific (TPM1-L185F) iPSC-CMs were treated with EMP or control vehicle for 5 days, followed by immunostaining for cardiac troponin T (TnT, red) and sarcomeric α-actinin (SAA, green), as well as confocal imaging. **C**, Representative 63x confocal images are shown; scale bar, 20 μm. **D**, Quantification of the sarcomere length using images described in **C**, analyzed by the radially averaged magnitude spectrµM (RAMS) method (*26*). Per group, *n* = 3 independent cardiac differentiations, *n* = 88 images per EMP-treated DCM iPSC-CMs (1.673 ± 0.011 μm), *n* = 88 images per CV-treated DCM iPSC-CMs (1.64 ± 0.009961 μm), one average data point per image; data are shown as mean ± SEM; **P < 0.01 (*P* = 0.0086; Mann-Whitney U test). **E-F**, The mitochondrial membrane potential as a parameter of mitochondrial function was quantified in DCM iPSC-CMs treated with EMP versus control vehicle. **E**, Representative images of DCM iPSC-CMs; live cell imaging after staining with the JC-1 dye, indicating monomers (green) and J-aggregates (red); images were taken with a 20x objective; scale bar, 50 μm; **F**, Quantification of the mitochondrial membrane potential based on detection of the JC-1 dye in DCM iPSC-CMs using a high-content platereader-based readout; per group, *n* = 3 independent experiments, *n* = 144 data points for EMP-treated DCM iPSC-CMs, *n* = 120 data points for CV-treated DCM iPSC-CMs, ***P < 0.0005 (*P* = 0.0003; Mann-Whitney U test); a.u., arbitrary units; data are shown as mean ± SEM. **G**, Mitochondrial Fe levels were measured in live DCM patient-specific (TPM1-L185F) iPSC-CMs treated with EMP versus control vehicle, using the FerroOrange dye. Per group, *n* = 3 independent experiments, *n* = 22 data points; **P < 0.005 (*P* = 0.0011; Mann-Whitney U test); a.u., arbitrary units; data are shown as mean ± SEM

As a molecular model of DCM, patient-specific iPSCs carrying the DCM mutation TPM1-L185F as well as healthy family control (WT) iPSCs were employed, which were characterized in detail before [19, 20]. DCM and WT iPSCs expressed regular levels of pluripotency markers (**S** Fig. 1) and following differentiation into iPSC-CMs using a well-established directed differentiation of monolayer cultures [15, 22–24], likewise, displayed standard expression of cardiac marker proteins (Fig. 1A, S Fig. 2). The DCM TPM1-L185F patient-specific iPSC-CMs were reported before to display reduced contractile function as well as diminished sarcomere length, compared to WT controls [19]. Therefore, firstly, the contractile and action potential (AP) properties of DCM patient-specific iPSC-CMs were assessed in comparison to WT controls (Fig. 1B-H). DCM TPM1-L185F iPSC-CMs displayed a significantly reduced contraction amplitude (peak height, Fig. 1B) as well as lower departure velocity (Fig. 1C), relative to WT controls. In line with this, optical mapping experiments revealed an increased AP duration (Fig. 1D-H, S Fig. 3A-B) in DCM TPM1-L185F iPSC-CMs versus WT controls. Moreover, as reported in detail earlier [19], we corroborated reduced mitochondrial function in DCM patient-specific iPSC-CMs, relative to WT controls (Fig. 1I-J). Next, we tested the effects of empagliflozin (EMP) treatment on DCM patient-specific iPSC-CMs, compared to control vehicle (Fig. 2). Following EMP treatment, the prolonged AP duration observed in DCM TPM1-L185F iPSC-CMs, compared to control vehicle, was significantly recovered (Fig. 2B-F). Moreover, DCM TPM1-L185F iPSC-CMs treated with EMP displayed improved sarcomere function, revealed by a significantly increased contraction amplitude (peak height, Fig. 2G) and departure velocity (Fig. 2H).

### EMP improves sarcomere contraction via recovering mitochondrial function and restoring mitochondrial Fe levels

Previously, DCM patient-specific iPSC-CMs carrying inherited DCM mutations in sarcomere proteins were found to display reduced sarcomere length and diminished sarcomere organization, compared to WT controls [15, 19, 25]. Therefore, we speculated whether EMP treatment would recover, in addition to sarcomere contractility, also the disrupted alignment of sarcomeres in DCM patient-specific iPSC-CMs. To explore this further, DCM TPM1-L185F iPSC-CMs were exposed to EMP for a duration of 2 days. Indeed, 48 h of EMP treatment resulted in a significantly improved sarcomere length of DCM TPM1-L185F iPSC-CMs, compared to control vehicle (Fig. 3A-B) as quantified via a previously reported method [15, 19, 25]. These experiments corroborated the EMP-mediated recovery of the sarcomere length in DCM TPM1-L185F iPSC-CMs, compared to control vehicle. Of note, the restoration of the sarcomere length in DCM TPM1-L185F iPSC-CMs was more pronounced following prolonged treatment of EMP for 5 days (Fig. 3C-D). We confirmed this finding using an independent approach for analysis of sarcomere length and sarcomere organization [26] (Fig. 3D).

Next, we sought to investigate a further potential basis for EMP-based recovery of sarcomere organization and function in DCM TPM1-L185F iPSC-CMs. Previously, reduced mitochondrial function has been reported in iPSC-CMs carrying inherited DCM mutations in sarcomere proteins [17, 19, 20]. Impaired mitochondrial function results in depleted ATP production and limits supply of ATP to sarcomeres, which entail a particularly high ATP demand due to their constant contractile function [19, 20]. Therefore, we investigated whether EMP treatment might recover mitochondrial function in DCM patient-specific iPSC-CMs. Indeed, EMP significantly improved the mitochondrial membrane potential of DCM TPM1-L185F iPSC-CMs (Fig. 3E-F).

**Fig. 4:**
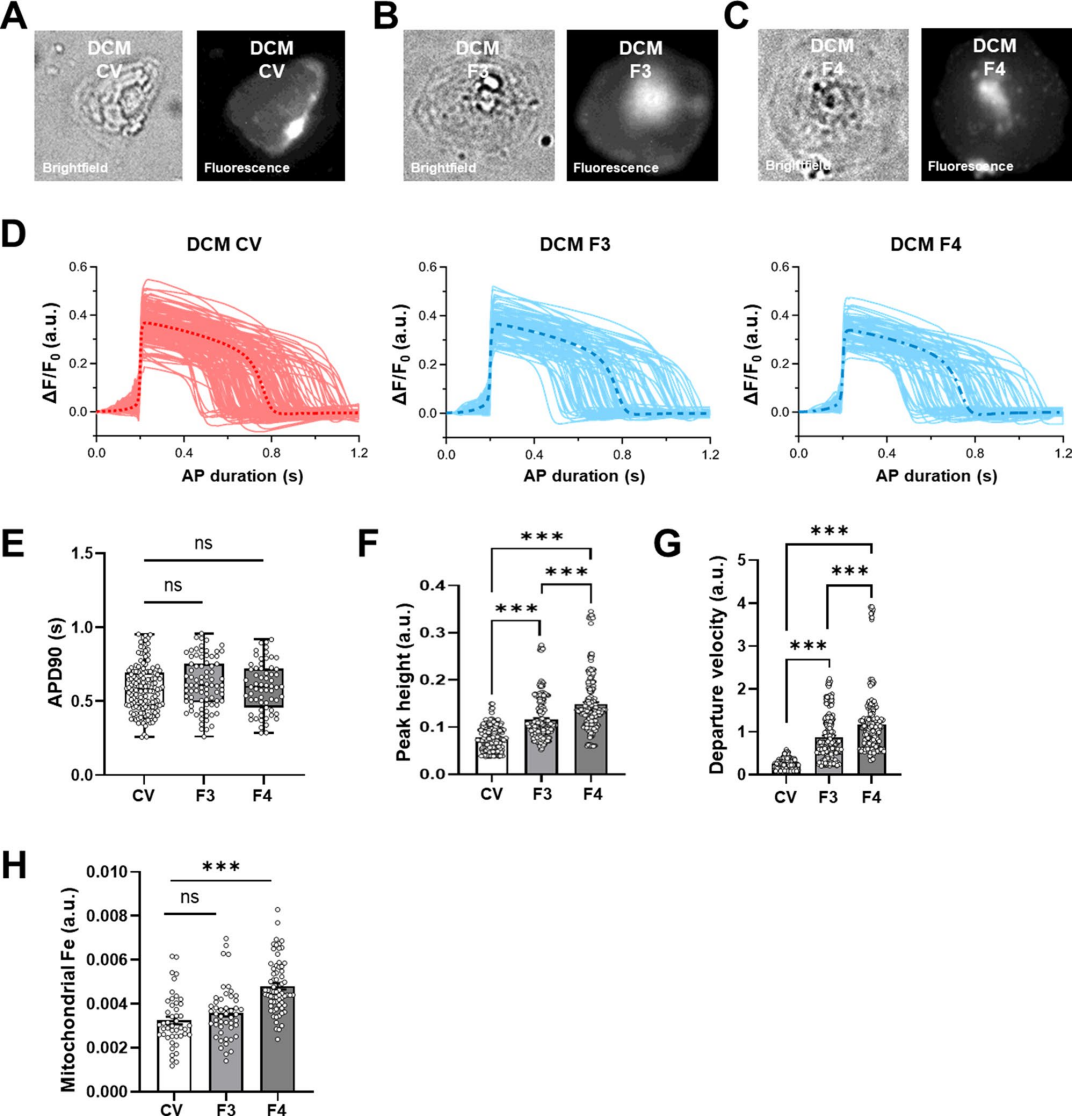
Treatment with a state-of-the-art pharmacological therapy for heart failure, F4, but not F3 improves AP properties and sarcomere function of DCM iPSC-CMs. **A-E**, Optical mapping of DCM TPM1-L185F iPSC-CMs treated with control vehicle (*n* = 150), F3 (*n* = 82), or F4 (*n* = 63). The control vehicle data group corresponds to the one shown in Fig. 2B. **A-C**, Representative images of recorded single-cell DCM iPSC-CMs treated with control vehicle (**A)**, F3 (**B**), or F4 (**C**) and loaded with the voltage-sensitive dye, FluoVolt, in brightfield mode (left) and fluorescence mode (right). **D**, Action potential (AP) duration for DCM patient-specific iPSC-CMs treated with control vehicle (left, data shown in this panel correspond to the data shown in Fig. 2D), F3 (middle), or F4 (right); **E**, AP duration (APD) 90, ns, statistically not significant (Kruskal-Wallis test and Dunn’s multiple comparison test). Data are shown as mean ± SEM; **F-G**, Contractility analysis of patient-specific DCM iPSC-CMs treated with the F3 or F4 drug combinations (*n* = 3 independent cardiac differentiations, each *n* = 10 recordings, F3 *n* = 245, F4 *n* = 231) versus control vehicle (*n* = 2 independent cardiac differentiations, each *n* = 15 recordings, *n* = 193 transients) using the IonOptix motion-traction platform. Data are shown as mean ± SEM; a.u., arbitrary units. **F**, Peak height of DCM iPSC-CMs, ***P < 0.0001 (***P < 0.0001; Kruskal-Wallis test); **G**, Departure velocity (speed to peak) of DCM iPSC-CMs; ***P < 0.0001 (*P* < 0.0001; Kruskal-Wallis test). **H**, Mitochondrial Fe levels were improved following treatment of DCM patient-specific iPSC-CMs with F4, but not F3, compared to control vehicle. Measurements were performed using the FerroOrange dye; per group, *n* = 2 independent experiments with CV and F3, *n* = 113 data points, and F4, *n* = 145 data points. CV versus F4, ***P < 0.0001 (*P* < 0.0001); CV versus F3, not statistically significant (ns) via Kruskal-Wallis multiple comparison test. a.u., arbitrary units. Data are shown as mean ± SEM

To address the molecular mechanism by which EMP may trigger improvement of the depressed mitochondrial function, we considered that in DCM iPSC-CMs, clathrin-mediated endocytosis (CME)-dependent signaling is disrupted [19, 20]. CME is the main uptake route of cargo critically required for basic cellular activities, such as transferrin-bound iron (Fe), which is important particularly for mitochondrial function. Impaired CME results in depleted mitochondrial Fe levels in DCM cardiomyocytes, as described before [19]. Restoring CME clearly recovered mitochondrial Fe levels as well as mitochondrial function, resulting in increased delivery of ATP, thereby positively modulating sarcomere function in DCM iPSC-CMs [19]. Indeed, we also confirmed that inhibition of CME via blocking the PIP5K1C kinase, which prevents an early step in CME, PI4,5P2-dependent clathrin-coated pit formation, negatively affects sarcomere organization (**S** Fig. 4A-B). We thus speculated that EMP treatment might improve CME in cardiomyocytes, thereby recovering mitochondrial Fe levels. We investigated mitochondrial Fe levels using a previously reported assay [19] and found that EMP significantly improved mitochondrial Fe in DCM TPM1-L185F iPSC-CMs, compared to control vehicle (Fig. 3G).

Overall, our results showed a statistically significant recovery in sarcomere length following EMP treatment in DCM TPM1-L185F iPSC-CMs. Thus, these results indicate that in DCM patient-specific iPSC-CMs, restoring mitochondrial Fe levels and mitochondrial function could lead to an improved sarcomere length and organization. This is in line with previous reports on drug-dependent improvement of CME-dependent signaling in DCM CMs [19].

### A state-of-the-art HF therapy including EMP improves the sarcomere contractile dysfunction and mitochondrial Fe levels in DCM iPSC-CMs

In patients with heart failure and reduced ejection fraction (HFrEF), EMP is typically administered in a combination therapy containing four medications, such as eplerenone, sacubitril/valsartan, and metoprolol, together with EMP, referred to as “fantastic four” (F4) [21].

Thus, we next investigated the consequences of this state-of-the-art combinatorial therapy, including EMP, at the molecular level, regarding sarcomere function and organization as well as mitochondrial Fe levels. We quantified the effects of the F4 combination of drugs (eplerenone, sacubitril/valsartan, metoprolol, and EMP) compared to F3 (eplerenone, sacubitril/valsartan, and metoprolol) or control vehicle in DCM patient-specific iPSC-CMs (Fig. 4). While the action potential duration (APD90) showed a trend to be increased in the presence of the F3 or F4 drug combinations, respectively, this difference was not statistically significant, compared to control vehicle (Fig. 4A-E). However, we found the contractile properties of DCM TPM1-L185F iPSC-CMs recovered in the presence of the F3 drug combination, compared to control vehicle (Fig. 4F-G). Of note, the rescue of sarcomere contraction was significantly higher in the presence of the F4 drug combination than the F3 drug combination (**Fig. F-G**). In line with this, treatment with F4, but not F3, improved the mitochondrial Fe levels of DCM TPM1-L185F iPSC-CMs, compared to control vehicle (Fig. 4H). Together, these results confirm that EMP is a crucial part of the current state-of-the-art HFrEF therapy, leading to beneficial molecular effects via different signaling pathways. Specifically, these findings support that a state-of-the-art pharmacological HFrEF therapy, including EMP, partially recovers sarcomere dysfunction via restoring aspects of CME-dependent signaling, resulting in improved mitochondrial Fe levels.

### EMP restores ventricular electrophysiological properties in a translational large-animal model of AF-induced cardiomyopathy

To validate the cellular findings in an in-vivo translational setting, a large-animal model of atrial fibrillation (AF) complicated by heart failure (HF) was employed. Domestic pigs were randomized to sinus rhythm control (Ctrl), AF/HF with control vehicle (HF + CV), or AF/HF treated with empagliflozin (HF + EMP). AF was induced by device-based atrial burst pacing without atrioventricular nodal ablation, allowing for rapid ventricular rate conduction and the development of arrhythmia-induced cardiomyopathy.

**Fig. 5:**
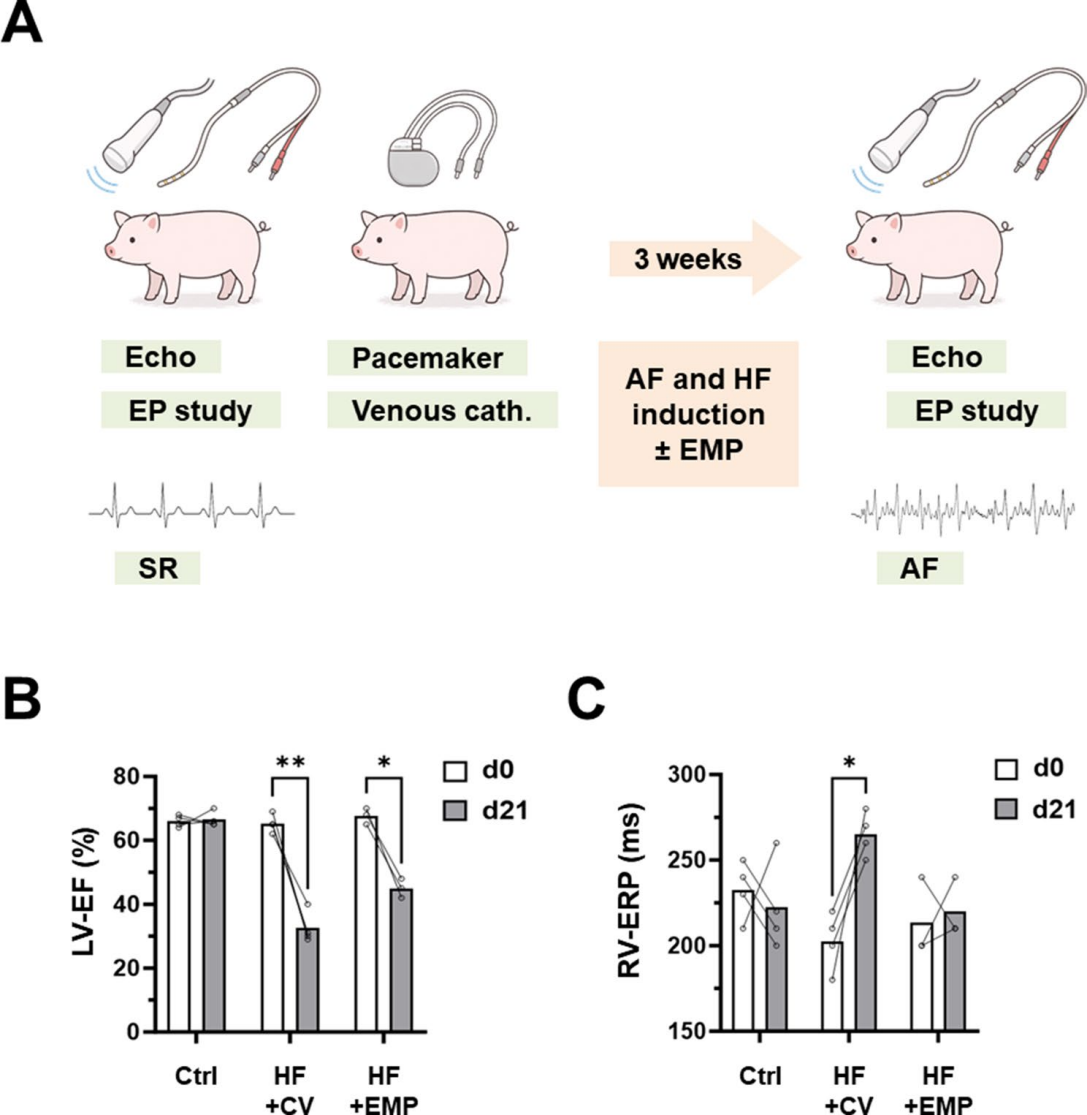
Empagliflozin (EMP) restores pathological ventricular effective refractory period prolongation in a translational porcine model of atrial fibrillation and heart failure. **A**, Schematic overview of the experimental protocol. Following baseline echocardiographic and electrophysiological (EP) assessment, domestic pigs (35–50 kg) underwent pacemaker and central venous catheter implantation and were randomized to a sinus rhythm control (Ctrl) group receiving control vehicle (CV) or to groups with atrial fibrillation (AF) and heart failure (HF) induction receiving empagliflozin (EMP) or CV. After a 3-week experimental period with daily intravenous EMP or CV administration, echocardiographic and EP studies were repeated. **B**, Left ventricular ejection fraction (LV-EF) determined by echocardiography before and after AF and HF induction with CV or EMP treatment. *P < 0.05 (*P* = 0.028) and **P < 0.01(*P* = 0.009) by two-tailed paired Student’s t-tests adjusted by Holm–Bonferroni correction. **C**, Right ventricular effective refractory periods (RVERP) measured during EP studies using S1–S2 stimulation protocols in the three experimental groups. *P < 0.05 (*P* = 0.042) by two-tailed paired Student’s t-tests adjusted by Holm–Bonferroni correction. Data are shown as mean ± SEM of *n* = 3–4 pigs per group

Echocardiographic assessment demonstrated preserved left ventricular ejection fraction (LV-EF) in control animals, whereas AF/HF induction resulted in a marked reduction of LV-EF in HF + CV animals. EMP treatment attenuated this decline, with LV-EF partially preserved compared with HF + CV (Fig. 5B). Within-group paired analyses revealed no significant change in LV-EF in control animals, while both AF/HF groups exhibited a significant reduction after AF/HF induction, which was less pronounced under EMP treatment. Electrophysiological studies further revealed substantial alterations in ventricular refractoriness. Right ventricular effective refractory periods (RV-ERP) remained stable in control animals but were significantly prolonged following AF/HF induction in the HF + CV group, consistent with electrical remodeling associated with arrhythmia-induced cardiomyopathy (AiCM). In contrast, EMP treatment significantly mitigated RV-ERP prolongation, resulting in shorter ventricular refractory periods compared with HF + CV and values closer to those observed in control animals (Fig. 5B). Therefore, these in-vivo findings demonstrate that EMP favorably modulates ventricular electrical remodeling and systolic function in a clinically relevant large-animal model of AiCM, supporting the translational relevance of the EMP-mediated electrophysiological and contractile improvements observed in human DCM TPM1-L185F iPSC-derived cardiomyocytes.

Collectively, our findings suggest EMP recovers sarcomere function (Fig. 2G-H). We propose this to occur through improved mitochondrial Fe levels and mitochondrial function (Fig. 3E-G), in line with a restored CME-dependent signaling [19]. In vitro improvements in sarcomere function are in line with the partially preserved LV-EF in-vivo after EMP treatment. Moreover, recovered AP duration in iPSC-CMs are supported by shortened refractory periods observed in the treated porcine model.

**Fig. 6:**
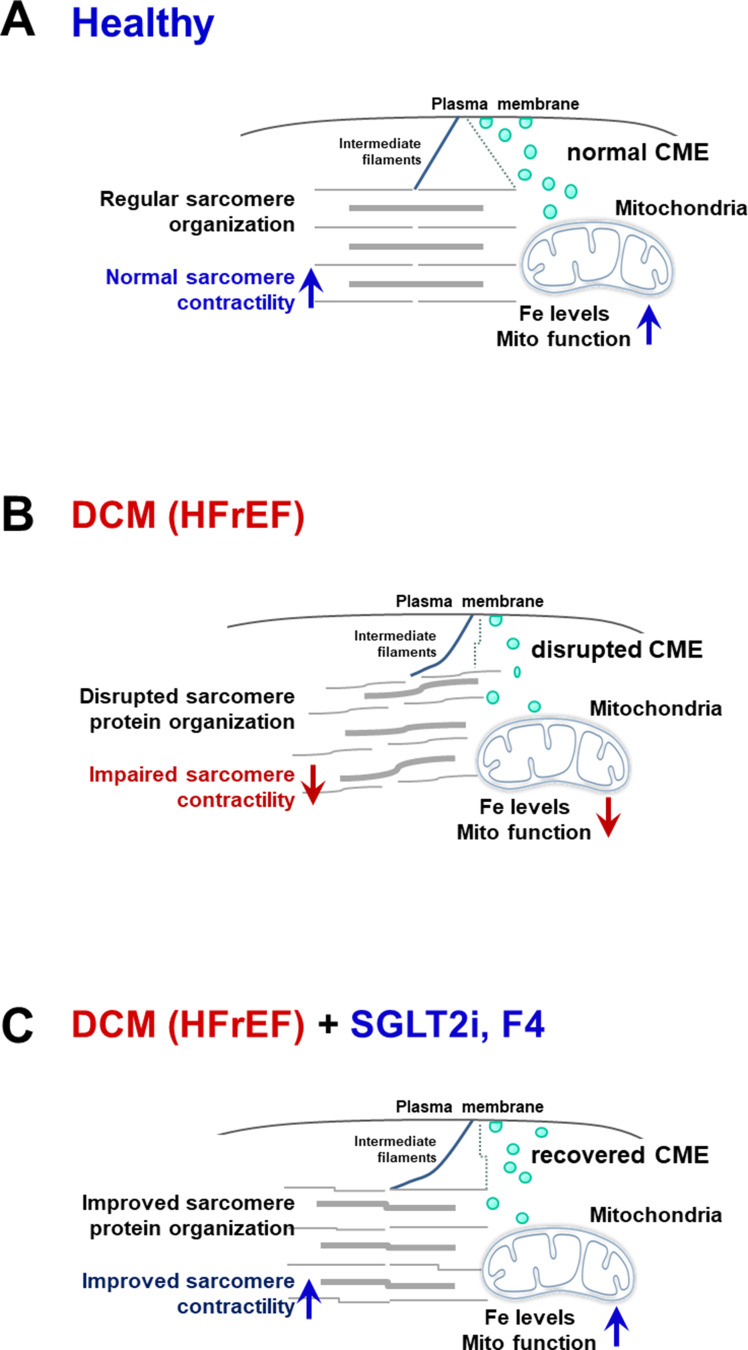
Schematic representation of additional SGLT2i mechanisms of action resulting in improved sarcomere function. **A-C**. SGLT2i function in CMs by several routes that improve sarcomere functions, such as contractility, for example by inhibiting the plasma membrane (PM)-localized Na⁺/H⁺ exchanger type 1 (NHE1), or phosphorylation of the SERCA-regulating protein phospholamban. An additional route for SGLT2i-dependent improvement of sarcomere functions may be the upregulation of defective CME-signaling in DCM CMs, resulting in recovered mitochondrial iron (Fe) levels. Consequently, this restores mitochondrial function and allows for more efficient ATP supply to sarcomeres, ultimately improving sarcomere function in an iPSC-CM model of DCM (HFrEF). This mechanism of action may also take place in combination treatments of eplerenone, sarcubitril/valsartan, metoprolol, and an SGLT2i, such as EMP (F4), which are standard therapeutic choices for patients with DCM and HFrEF. Intermediate filaments, connect sarcomeres and plasma membrane (PM) and adjacent branched actin network supporting formation of clathrin-coated pits (CCPs) at the PM. Blue circles, early endosomes budding from CCPs during CME at the PM

Of note, the CME-dependent signaling pathway can be modulated to some extend by F4 treatment, restoring partially the mitochondrial Fe levels, as well as electrophysiological and contractile parameters (Fig. 6C).

## Discussion

The favorable effects of SGLT2 inhibitors, such as EMP, on cardiomyocytes have been proposed to include the modulation of different signaling functions. However, these have not yet been studied in detail in molecular human in-vitro models of inherited dilated cardiomyopathy (DCM), a frequent cause of HFrEF. Here, we investigated these aspects in human patient-specific iPSC-CMs carrying an inherited mutation in a sarcomere protein, TPM1, which is prevalent in patients with familial DCM [19]. Previous studies reported SGLT2i to modulate various subcellular pathways, some of which may affect sarcomere organization of CMs directly. Examples are the SGLT2i-dependent increase of phospholamban phosphorylation at Ser16 [10], or increased phosphorylation of cardiac Troponin I (cTnI) and cardiac myosin binding protein C (cMyBP-C) [27–29]. But as other studies show, SGLT2 is expressed only minimally in CMs, indicating EMP to work through different mechanisms besides the known SGLT2 inhibition [30]. SGLT1 shows an overexpression in ischemia and diabetic cardiomyopathy in mice, indicating a relevant effect on the development of HF [31, 32]. However, EMP targets SGLT2 so specifically that off-target effects are unlikely to account for all treatment benefits of SGLT2 inhibition. A different signaling pathway modulated by SGLT2i is suppression of the Na⁺/H⁺ exchanger type 1 (NHE1) activity, which significantly attenuates symptomatic HF progression on systolic and diastolic dysfunctions [33]. EMP has been shown to target the sodium transporter NHE-1 directly, impairing NHE-1 flux, to decrease cytosolic sodium and Ca²⁺ [34, 35]. This results in an improved equilibrium of the Na⁺/Ca²⁺ exchanger (NCX), enhancing SERCA-mediated Ca²⁺ reuptake into the sarcoplasmic reticulum [36]. In iPSC-CMs carrying an inherited hypertrophic cardiomyopathy mutation, altered NXC activity, mediating improved Ca²⁺ handling, could be observed after a very short EMP treatment of 15 min [9]. In line with this, these mechanisms may contribute to the recovery of contractility observed in EMP-treated DCM TPM1-L185F iPSC-CMs as well.

Furthermore, in rats and human tissue samples of heart failure with preserved ejection fraction (HFpEF), EMP-induced shifts of metabolic functions have been shown. In this context, lower cytosolic reactive oxygen species (ROS) due to EMP treatment consequently reduced inflammation and oxidative stress levels [27]. In addition, SGLT2i were shown to deliver anti-inflammatory signaling in diabetic cardiomyopathy models, e.g. by targeting and limiting NLR family pyrin domain containing 3 (NLRP3) inflammasome activation [37], activating mTOR signaling [38] or the Nrf2/ARE signaling pathways [39]. Together, these beneficial signaling events lead to a decelerated progression of diabetic cardiomyopathy phenotypes.

Moreover, EMP showed a favorable effect on energy metabolism in diabetic cardiomyopathy murine heart tissue [40]. Here, EMP supported the energy consumption of diabetic cardiomyopathy cardiomyocytes via increased ATP levels through modulation of the ketone body metabolism [40]. It is possible that the above-mentioned functions of EMP in lowering ROS and reducing inflammation contribute to improved mitochondrial function in EMP-treated disease models. Alternatively, or in addition, EMP may improve mitochondrial respiration, i.e., ATP production, by other means.

Our results confirm that SGLT2i treatment results in modulation of intracellular signaling events that recover sarcomere functions in DCM TPM1-L185F iPSC-CM models. This was confirmed by contractility recordings as well as examination of the action potential (AP) properties via optical mapping. Moreover, the sarcomere length of DCM TPM1-L185F iPSC-CMs was improved following EMP treatment, further supporting that EMP can assist the reversal of molecular pathological dysfunctions in DCM CMs. Particularly, the benefit of prolonged treatment durations suggested these, to achieve more substantial improvements in sarcomere length and organization. Our results demonstrating improved sarcomere functions following EMP treatment are in line with other suggested functions of SGLT2i, such as ameliorating dysfunctions of ion channels and reducing arrhythmias in rat neonatal and human iPSC-derived cardiomyocytes under hypoxic conditions [10].

In DCM CMs carrying inherited sarcomere protein mutations, sarcomere dysfunction such as reduced contractility is primarily due to the disease-causing mutations, which has been studied in the past decades in detail [13–15, 20]. This included studies with CRISPR/Cas engineered, isogenic control iPSC-CMs in which the inherited DCM mutation had been corrected in patient-derived iPSCs (mutation-corrected) [17, 19] or, alternatively, introduced into WT iPSCs (mutation-introduced) [14, 17, 19, 20]. However, another driver of pathogenesis in DCM are additional signaling dysfunctions in DCM CMs that are triggered by sarcomere dysfunctions and result in pathological events which ultimately exacerbate the reduced contractility even further [15, 19, 20, 41, 42]. This has been shown e.g. for defective clathrin-mediated endocytosis (CME)-dependent signaling, which results in depleted mitochondrial Fe levels and consequently impaired mitochondrial function in DCM CMs [19]. Due to this, in turn, reduced mitochondrial function was shown to limit ATP supply to the sarcomeres, further restricting their contraction, which is already reduced due to the DCM-causing sarcomere protein mutations [19]. Further evidence for this mechanism, as well as its translational relevance demonstrated, that recovering defective CME-dependent signaling by genetic or chemical means restored the uptake and distribution of CME-dependent cargo (such as transferrin-bound Fe). Subsequently, improved mitochondrial Fe levels as well as -function, rescued in part the sarcomere contractile function of DCM iPSC-CMs [19, 20]. CME is the main route for uptake of Fe in cells, including CMs, in the form of transferrin-bound Fe, which is subsequently distributed to intracellular destinations, such as mitochondria, via early endosomes (EEs). Other Fe uptake routes, such as via the Divalent Metal Transporter 1 (DMT1) protein, do not significantly contribute to the uptake of transferrin-bound Fe, as experiments with a DMT1 inhibitor, ebselen, in DCM iPSC-CMs demonstrated [19]. As earlier studies showed, in CMs, modulation of CME is necessary and sufficient for altering mitochondrial Fe levels and, consequently, sarcomere function, in DCM CMs [19, 20].

Here, our findings suggest that SGLT2 inhibition results in improved mitochondrial function due to recovering the CME-dependent uptake of critical cargo, such as transferrin-bound Fe, which restores mitochondrial Fe levels in DCM iPSC-CMs. Thus, in patient-specific iPSC-CMs carrying DCM mutations, SGLT2i may enhance mitochondrial Fe levels via boosting CME, thereby increasing mitochondrial output and presumably adding to recovery of sarcomere functions, such as impaired contractility. Of note, this project focused only on several key phenotypes of the impaired CME-dependent signaling pathway in DCM CMs. The recovered mitochondrial function observed in this study in DCM TPM1-L185F iPSC-CMs treated with EMP is in line with earlier reports in other models [8, 9] and therefore suggests a positive modification of CME-dependent signaling by EMP treatment.

Importantly, the translational relevance of these mechanistic findings was further supported by a large-animal model of AF complicated by HF. In this clinically relevant porcine model of AiCM, sustained AF with preserved atrioventricular conduction resulted in ventricular systolic dysfunction and pronounced electrical remodeling, reflected by prolonged RV-ERP. EMP treatment attenuated both functional and electrophysiological deterioration, partially preserving LV-EF and limiting ventricular refractoriness prolongation. These in-vivo observations extend our cellular findings by demonstrating that EMP favorably modulates ventricular electrical remodeling under conditions of chronic tachyarrhythmia and HF, thereby providing an important translational link between subcellular mechanisms and whole-organ electrophysiological phenotypes.

To further investigate which of the well-known specific components of the CME-dependent signaling pathway [19, 20] SGLT2 inhibition affects, remains the focus of interest for future studies. Several CME-signaling components are potential targets for direct or indirect modulation by SGLT2 inhibition, e.g., actin scaffold formation and concomitant PI4,5P2 kinase function at the plasma membrane, which supports clathrin-coated pit (CCP) formation; early endosome (EE) budding from CCPs and their distribution within the CM; or delivery of critical EE cargo, such as transferrin-bound Fe, at key recipient organelles such as the mitochondria [19, 20]. Investigating these questions will be relevant topics of follow-up research.

Patients with HFrEF typically receive an SGLT2i in a combination therapy with e.g. a mineralocorticoid receptor antagonist (MRA) such as eplerenone; an angiotensin receptor blocker and neprilysin inhibitor (ARNI) such as sacubitril/valsartan; and a beta-blocker such as metoprolol. Therefore, our study evaluated this drug combination, referred to as “fantastic four” or F4 [21] compared to F3 (eplerenone, sacubitril/valsartan, metoprolol) in patient-specific iPSC-CMs in order to evaluate the effect of adding EMP to the F3 combination of drugs. Specifically, our results show that EMP contributes a substantial part of the rescue modalities delivered by an F4 treatment in DCM TPM1-L185F iPSC-CMs, versus control vehicle. Within the comparison of the F3 treatment to the F4 treatment, the addition of EMP in the F4 group is the only difference between the two group’s treatments. Therefore, we attribute observed effects to the addition of EMP in-vitro. Sarcomere function, reflected by specific contractile parameters and the sarcomere length, as well as the mitochondrial Fe levels were significantly recovered following F4 treatment in DCM iPSC-CMs. In comparison, the F3 combination of drugs without EMP could not deliver all of these improvements. Our findings are supported by clinical studies in HFrEF patients. For example, a marked increase in left ventricular ejection fraction (LVEF) has been observed in > 50% of patients treated with SGLT2i on top of the standard therapy, compared to > 11% showing this benefit with conventional therapy alone [43].

It should be noted that neither EMP nor F4 treatment recovered all investigated aspects of the pathological sarcomere dysfunction in DCM patient-specific iPSC-CM models. We found that the rescue of action potential properties did not extend to all parameters; for example, the AP duration following F4 treatment showed no significant change, compared with control vehicle. Furthermore, EMP and F4 recovered specific contractile properties of DCM TPM1-L185F iPSC-CMs, such as the peak height and the departure velocity. However, not all aspects of pathological dysfunction in the contractile performance of DCM TPM1-L185F iPSC-CMs were recovered by EMP or F4 treatments. Prolonged culture of iPSC-CMs might allow to observe stronger effects of EMP treatment on other contractile parameters, as found in an iPSC-derived HCM model [9]. Moreover, in this context, the limitations of in-vitro culture models should be considered, compared to clinical trials in human patients. The bioavailability of the drugs delivered via the circulatory system as well as to the heart as an entire organ stipulates much higher uses of drug concentrations in HFrEF patients, than used in typical in-vitro cell-based studies. Moreover, when calculating clinically relevant concentrations, sacubitril/valsartan (ARNI) and beta-blockers such as metoprolol would be given at a > 10x higher concentration than the other drugs, downscaling blood concentrations in treated patients to the in-vitro model [44, 45]. Therefore, the iPSC-CMs would be exposed to an imbalanced drug dose. Moreover, scaled down in-vivo concentrations of ARNI and beta-blockers would have corresponded to circa 400 µM each for the corresponding downscaled in-vitro dose. As all F4 drugs are dissolved in DMSO for in-vitro drug treatment, the biochemically high concentrations of DMSO would have been toxic to the in-vitro cell culture [46, 47]. Thus, the fully scaled-down concentration of ARNI or beta-blocker would have made the experimental measurements invalid. Because we chose an EMP concentration of 5 µM in iPSC-CMs, we set the maximal relative total concentration of drugs (F4) or control vehicle in the iPSC-CM based system to 5 µM to avoid DMSO-induced cell toxicity [46, 47]. Thus, in-vitro based studies in DCM patient-specific iPSC-CMs are indicative for detailing further mechanistic and functional insight into the molecular action of these drugs yet are compromised by an indirect and relative relation to their pharmacological effects observed in HFrEF patients. This is a clear limitation of our cellular in-vitro DCM iPSC-CM model system. Previous studies performed on animal models and patients could employ oral or intravenous drug distribution and therefore higher concentrations as well as longer treatments [48–50]. Thus, in the setting of our in-vitro model of DCM iPSC-CM, a less intense effect of the combination including ARNI and beta-blocker could be due to the in-vitro treatment approach employed here, demanding lower drug doses to avoid toxicity. Moreover, future studies should investigate the consequences of individual treatments of ARNI, beta-blocker, or MRI on CME-dependent signaling in DCM CMs, compared to the F3 or F4 drug combinations. This would extend insights into the molecular functions of these drugs, particularly regarding any effect on CME signaling.

Together, our findings further revealed molecular mechanistic effects of SGLT2 inhibition and the “fantastic four” state-of-the-art therapy, supporting that these benefits are exerted via targeting of different molecular signaling pathways, including CME-dependent signaling. Onward, the results of this study could be further confirmed in other models, e.g., in live adult patient-derived cardiomyocytes from surgical leftovers, which were reported to recapitulate active CME-dependent signaling [19]. Showing improvement of mitochondrial Fe levels in this model would corroborate the present study in another clinically relevant system, further adding to mechanistic understanding of the subcellular signaling events modulated by SGLT2 inhibition.

Overall, the present study may benefit different types of future mechanistic studies, but also disease modelling for DCM due to inherited mutations in sarcomere proteins. Thus, we consider our approach a starting point for modelling the molecular signaling functions of SGLT2 inhibition in DCM cardiac cells. In this context, our study addressed the purpose of further elucidating subcellular defects in presence of the DCM TPM1-L185F mutation and identifying palpable means to rescue molecular patho-phenotypes in DCM. Thereby, the present findings could support further personalized treatment regiments in a severe cardiac disease, DCM.

## Materials and methods

Further details can be found in the supplementary materials.

### Ethics approval and consent to participate

All protocols for studies with human iPSC were approved by the Goettingen University Ethical Board (15/2/20, 20/9/16An, and 7/5/24) and the Odense University Ethical Board (Projekt ID S-20140073HLP). Informed consent was obtained from all participants, and all research was performed in accordance with relevant guidelines and regulations. The procedures used in this study adhere to the tenets of the Declaration of Helsinki.

All animal procedures complied with internationally accepted standards for laboratory animal care, including the Guide for the Care and Use of Laboratory Animals of the US National Institutes of Health, EU Directive 2010/63/EU, the current German Animal Welfare Act, and the ARRIVE guidelines. Experimental protocols were reviewed and approved by the responsible local animal ethics authority (Regierungspräsidium Karlsruhe, Germany, approval no. G-229/21 and G-131/22).

### Generation, culture, and cardiac differentiation of human iPSCs

WT iPSCs were derived and characterized as described before [19, 20]. Matrigel-coated plates (Corning) were used to culture human iPSCs utilizing chemically defined E8 medium as reported previously [12, 24]. Culture medium was changed daily and iPSCs were passaged every three to four days via EDTA (Life Technologies) [12, 24]. To differentiate iPSCs into beating iPSC-CMs, a small molecule-based approach was used as reported previously [15, 22, 23, 51, 52]. On day 20–25 of cardiac differentiation, the beating iPSC-CMs were dissociated using trypleE (Life Technologies) and plated in the appropriate assay formats for experiments. Further, the human iPSC-CMs were cultured in RPMI (Life Technologies) with B27 supplement (Life Technologies) until use for experiments and analysis.

### Contractility analysis

Human iPSC-CMs were plated into matrigel-coated 6-well-plates in monolayers and, after one day of recovery, treated with 5 µM DMSO (Sigma-Aldrich) or 5 µM EMP (Selleckchem BI 10773). A DMK 33UX174 camera and Axio Observer A1 (Zeiss) microscope with Ion Optix software and were used and a first analysis of the recordings performed with CytoSolver software as mentioned by the vendor. Further analysis was done as reported [53–56]. Pixel intensity measurements were used to collect values for departure velocity, return velocity, peak height, time to peak 90% and time to baseline 90%. Contraction duration was calculated by: Time to peak - Time to peak 10% + Time to baseline 90% for each transient.

### Fe measurements

Human iPSC-CMs were harvested and plated into matrigel-coated 96well plates and, after one day of recovery, cultured in RPMI (Life Technologies) with B27 supplement (Life Technologies) including drug treatments where indicated. After incubation with Mitotracker Green (Cell Signaling Technology) at 37 °C as by the manufacturer´s instructions, cells were incubated with FerroOrange (Dojindo EU GmbH) as well as with the nuclear dye Hoechst (Thermo Fisher Scientific) according to the manufacturer´s instructions and as described before [19]. Fluorescence was analyzed via a Cytation 3 reader (BioTek) and mitochondrial Fe levels were analyzed normalizing reads for relative mitochondrial content (Mitotracker) and cell numbers (Hoechst) as reported before [19].

### Mitochondrial membrane potential

Human iPSC-CMs were harvested and plated into matrigel-coated 96well plates and, after one day of recovery, cultured in RPMI (Life Technologies) with B27 supplement (Life Technologies) including drug treatments where indicated. The mitochondrial membrane potential was measured using the JC1 dye kit (Thermo Fisher Scientific). The kit was used according to the manufacturer´s instructions and cells were incubated with the dye for 30 min at 37 °C. Fluorescence readout was performed in a Cytation 3 reader (BioTek) [19].

### Optical mapping

Human iPSC-CMs were plated into matrigel-coated 6well plates and, after one day of recovery, treated for 48 h with 5 µM CV (DMSO, Sigma-Aldrich) or 5 µM EMP (Selleckchem BI 19773). Optical mapping experiments were performed using an Olympus BX51WI upright microscope equipped with a 20× water immersion objective. iPSC-CMs were incubated with the voltage-sensitive dye FluoVolt™ (Thermo Fisher Scientific) according to the manufacturer’s instructions. Cells were maintained at a constant temperature of 37 °C using a dual automatic temperature controller (Warner Instruments, TC-344B) connected to the recording chamber. For electrical pacing, field stimulation was delivered via platinum electrodes connected to a stimulus isolator (World Precision Instruments, A365). Cardiomyocytes were paced at 1 Hz, with stimulus amplitude adjusted to ensure consistent capture. Optical recordings were acquired with an sCMOS camera (Andor Zyla 4.2) at 200 frames per second (fps). Camera acquisition was synchronized with the pacing protocol, and fluorescence emission was collected through the appropriate filter set for FluoVolt. Recorded image sequences were processed with a custom-built Python program to convert raw fluorescence images into single-cell optical traces. A machine learning–based classification (MLS) approach was then applied to the extracted traces to categorize cardiomyocytes based on electrophysiological phenotype. The resulting action potential waveforms were subsequently analyzed in Igor Pro 9 (WaveMetrics) using custom-written functions to extract quantitative electrophysiological parameters, including action potential amplitude, upstroke rising time, and action potential duration (APD).

### Large animal model of atrial fibrillation (AF) complicated by heart failure (HF)

The 11 male domestic pigs (bodyweight 35–50 kg) included in this study were housed under specific pathogen–free conditions at 20 ± 2 °C with stocking densities compliant with Directive 2010/63/EU. A 12-h light/dark cycle was maintained. Animals had free access to water and were fed twice daily with a standardized complete diet (SAF 130 M, ZG Raiffeisen, Karlsruhe, Germany). Environmental enrichment included wooden chew blocks, chains, and feeding balls.

Sedation was achieved using azaperone (Elanco, Bad Homburg, Germany), midazolam (Hameln Pharma Plus, Hameln, Germany), and ketamine (Zoetis Deutschland, Berlin, Germany). General anesthesia was induced with propofol (Fresenius Kabi, Bad Homburg, Germany), and perioperative analgesia was provided with buprenorphine (Bayer Vital Tiergesundheit, Leverkusen, Germany). Transthoracic echocardiography was performed in spontaneously breathing anesthetized animals. Left ventricular ejection fraction (LV-EF) was assessed by visual estimation in modified long-axis, two-chamber, and four-chamber views using a Vivid E9 system (GE Germany GmbH, Munich, Germany) by an experienced investigator.

Jugular venous access was obtained using the Seldinger technique. Under fluoroscopic guidance, quadripolar electrophysiology catheters were positioned at the superior vena cava–right atrial junction and in the right ventricular apex. Intracardiac pacing was delivered using a UHS 20 stimulus generator (Biotronik, Berlin, Germany), and electrocardiograms were recorded and analyzed using the Bard Electrophysiology Clearsign system (Bard Electrophysiology Division, Lowell, MA, USA). Pacing thresholds ranged from 0.5 to 3 V at 2.9 ms, with stimulation applied at twice the diastolic threshold. Right ventricular effective refractory periods (RV-ERP) were assessed using a standard extrastimulus protocol consisting of eight basic stimuli (S1) at a cycle length of 400 ms, followed by a premature stimulus (S2) decremented in 10-ms steps until loss of ventricular capture. RV-ERP was defined as the first S1–S2 interval failing to elicit a propagated ventricular response.

After completion of echocardiographic and electrophysiological assessments, anesthesia was maintained with isoflurane (Baxter Deutschland GmbH, Heidelberg, Germany). Dual-chamber pacemakers (Abbott Medical, Eschborn, Germany) were implanted as reported earlier [57]. In brief, after creating an intramuscular pocket in the region of the lateral neck muscles, venous access was obtained via the jugular vein using the Seldinger technique. Active fixation pacemaker leads (CapSureFix 5076, Medtronic, Dublin, Ireland) of 52 cm and 58 cm length were placed in the RA appendage and the apicoseptal region of the RV under radiographic control using two 7 F splitable introducers sheaths (Abbott). After stimulation threshold (< 1 V at 0.4 ms), impedance (300–1000 ohm), and sensing (> 1.5 mV in the atrium and > 5 mV in the ventricle) were confirmed by the PSA function of the Abbott Merlin pacemaker interrogator, pacemaker leads were sewed to the muscular fascia with three 0-gauge braided non-absorbable sutures each. After connecting both leads to an Abbott Ellipse device, programmed with a proprietary AF-induction firmware, careful hemostasis, antibiotic irrigation of the device pocket with cefuroxime, and layered wound closure were performed.

Atrial fibrillation was induced by atrial burst pacing using a device-based biofeedback algorithm as reported [58]. High-frequency pacing (40 Hz for 30 s) was delivered to the right atrium, followed by a 20-s observation period to assess arrhythmia persistence. Burst pacing was repeated upon spontaneous conversion to sinus rhythm and inhibited once sustained AF was established, allowing controlled and reproducible AF induction. No atrioventricular nodal ablation was performed, permitting rapid ventricular rate conduction during AF and resulting in the development of arrhythmia-induced cardiomyopathy (AiCM).

Central venous catheters were implanted via the left jugular vein using the Seldinger technique: A five-lumen polyurethane catheter (Teleflex inc., Wayne, Pennsylvania, USA) was advanced into the superior vena cava under fluoroscopic guidance, with correct positioning confirmed by fluoroscopy and venous blood aspiration. The catheter was tunneled subcutaneously to the neck of the animal, secured with non-absorbable sutures, and covered with a sterile dressing.

Empagliflozin was obtained from Biozol (Eching, Germany), dissolved in DMSO at a concentration of 90 mg/mL, and stored at − 20 °C. Animals received daily intravenous empagliflozin at a dose of 3 mg/kg body weight via the central venous catheter or an equivalent volume of control vehicle (DMSO).

Perioperative antibiotic prophylaxis with cefuroxime (750 mg intramuscularly) and postoperative analgesia with buprenorphine (intramuscular, titrated to effect) were continued for 48 h. At the end of the 21-day experimental period, pigs were sedated as described, and echocardiography as well as EP assessment was repeated. Finally, animals were euthanized under deep anesthesia by intravenous administration of potassium chloride (40 mL, 1 mol/L).

### Statistical analysis

To determine if the data followed a normal distribution, a Shapiro-Wilk test was conducted. When both datasets were normally distributed, an unpaired Student’s t-test was applied to assess statistical significance. If normality was not met for either of the independent datasets, a non-parametric test such as a Mann-Whitney test or a Kruskal-Wallis test was used instead. Unless indicated, the number of independent cardiac differentiations equals the number of independent experiments performed. P-values < 0.05 were considered statistically significant. Data are presented as mean ± standard error of mean (SEM).

## Supplementary Information

Below is the link to the electronic supplementary material.

Supplementary Material 1

## Data Availability

Because of the sensitive nature of the data collected for this study, the datasets collected during the current study are available from the corresponding author on reasonable request.
