# Supplementary material for: SGLT2 inhibition improves sarcomere contractile dysfunction in human models of dilated cardiomyopathy
Source: J Transl Med. 2026 Jul 31;24:1056. doi: 10.1186/s12967-026-08702-0 (PMC13474845; doi:10.1186/s12967-026-08702-0)
Supplement: Supplementary file 1 [file 12967_2026_8702_MOESM1_ESM.pdf]

SUPPLEMENTAL MATERIALS

SGLT2 inhibition improves sarcomere contractile dysfunction in human models of dilated cardiomyopathy

Daria Plota[1,2#], Hafiza Nosheen Saleem[1,2#], Kun-Han Lin[3], Ruheen Wali[1,2], Cleophas Cheruiyot[1,2], Luca Münzel[1,2], Tabea Hutschenreiter[1,2], Malte Tiburcy[2,4], Tim Meyer[2,4], Michael Habeck[5], Felix Wiedmann[1,2], Constanze Schmidt[1,2], Wolfram-Hubertus Zimmermann[2,4,6], Antje Ebert[1,2,6]*

[1]Heart Research Center, Department of Cardiology and Pneumology, University Medical Center, Göttingen University; [2]DZHK (German Center for Cardiovascular Research), partner site Göttingen, Germany; [3]Laboratory of Membrane Biophysics, Max Planck Institute for Multidisciplinary Sciences, Göttingen, Germany; [4]Institute of Pharmacology and Toxicology, University Medical Center Göttingen, Georg August University, Göttingen, Germany; [5]Microscopic Image Analysis Group, University Medical Center Jena, Jena, Germany; [6]Cluster of Excellence 'Multiscale Bioimaging: from Molecular Machines to Networks of Excitable Cells' (MBExC), University of Göttingen, Göttingen, Germany.

SUPPLEMENTAL MATERIALS AND METHODS

Immunostaining and confocal imaging. Human iPSCs were plated on coverslips, fixed with 4% PFA and permeabilized with 0.3% TritonX-100 at RT. Subsequently, iPSCs were incubated with primary antibodies: Oct3/4 (R&D Systems), TRA-1-81 (DSHB), SOX2 (Novus), SSEA4 (R&D Systems), DAPI (Sigma) at 4°C. Secondary antibodies (Alexa Fluor dyes, Thermo Fisher Scientific) were incubated at RT.

Human iPSC-CMs were plated on coverslips (Thermo Fisher Scientific) and fixed with 4% PFA. 0.2% TritonX-100 was used for permeabilization at room temperature (RT) and incubation with primary antibodies: TnT (Thermo Fisher Scientific), SAA (Sigma), MYBPC3 (Abcam) and MYH7 (Proteintech) at 4 °C followed. Secondary antibodies (Alexa Fluor dyes, Thermo Fisher Scientific) were incubated at room temperature.

Afterwards, all coverslips were mounted onto glass slides and a confocal microscope (LSM 710 Meta, Zeiss), equipped with a 63x oil-immersed objective, was used to perform imaging, as previously described (*1*-*3*).

Drug treatments. Between day 20 to 22 of differentiation, human iPSC-CMs were harvested and plated in desired format for experiments. After one day of recovery in RPMI (Life Technologies) with B27 supplement (Life Technologies), cells were treated for the indicated time periods. EMP treatment was performed using EMP (Selleckchem BI 10773) dissolved in DMSO (Sigma-Aldrich). Control vehicle (CV) was DMSO. Cells were treated with 5 µM EMP (Selleckchem BI 10773) or CV, diluted in RPMI (Life Technologies) with B27 supplement (Life Technologies) (*4*). The PIP5Ks-inhibitor UNC3230 (MedChemExpress) was treated in iPSC-CMs for 48 h, as described before (*5*). For treatment with F4, cells were treated for 2-5 days with eplerenone (MedChemExpress, HY-B0251), sarcubitril/valsartan, sold under the brand name entresto (MedChemExpress, HY-18204A), metoprolol (MedChemExpress, HY-17503) and EMP (Selleckchem BI 10773), 1.25 µM each, all together 5 µM, diluted in RPMI (Life Technologies) with B27 supplement (Life Technologies). F3 treatment used the drugs Eplerenone, Entresto and Metoprolol, each 1.25µM, all together 3.75 µM, diluted in RPMI (Life

Technologies) with B27 supplement (Life Technologies). All drugs were dissolved in DMSO; 5 μM DMSO was used as CV (*6-8*).

Sarcomere length analysis. Between day 20 to 24 of differentiation, human iPSC-CMs were harvested and plated on coverslips. After recovery for 1 to 2 days, treatment with 5μM DMSO or 5μM EMP was given as described. Fixation was done in 4% paraformaldehyde and staining with primary antibodies against cardiac troponin T (rabbit; Thermo Fisher Scientific) and sarcomeric alpha-actinin (mouse, Sigma-Aldrich) overnight. As secondary antibodies, anti-Mouse IgG Alexa Fluor 568 and anti-Rabbit IgG Alexa Fluor 488 were used to stain coverslips. At a confocal LSM710 microscope, a 63x objective (oil-immersed) was used to acquire images for the relevant channels. ImageJ was used for image analysis according to our previously described methods (*2, 5, 9*) where indicated. Alternatively, sarcomere length was analyzed via the radially averaged magnitude spectrμM (RAMS) method as reported (*10*).

SUPPLEMENTAL FIGURES

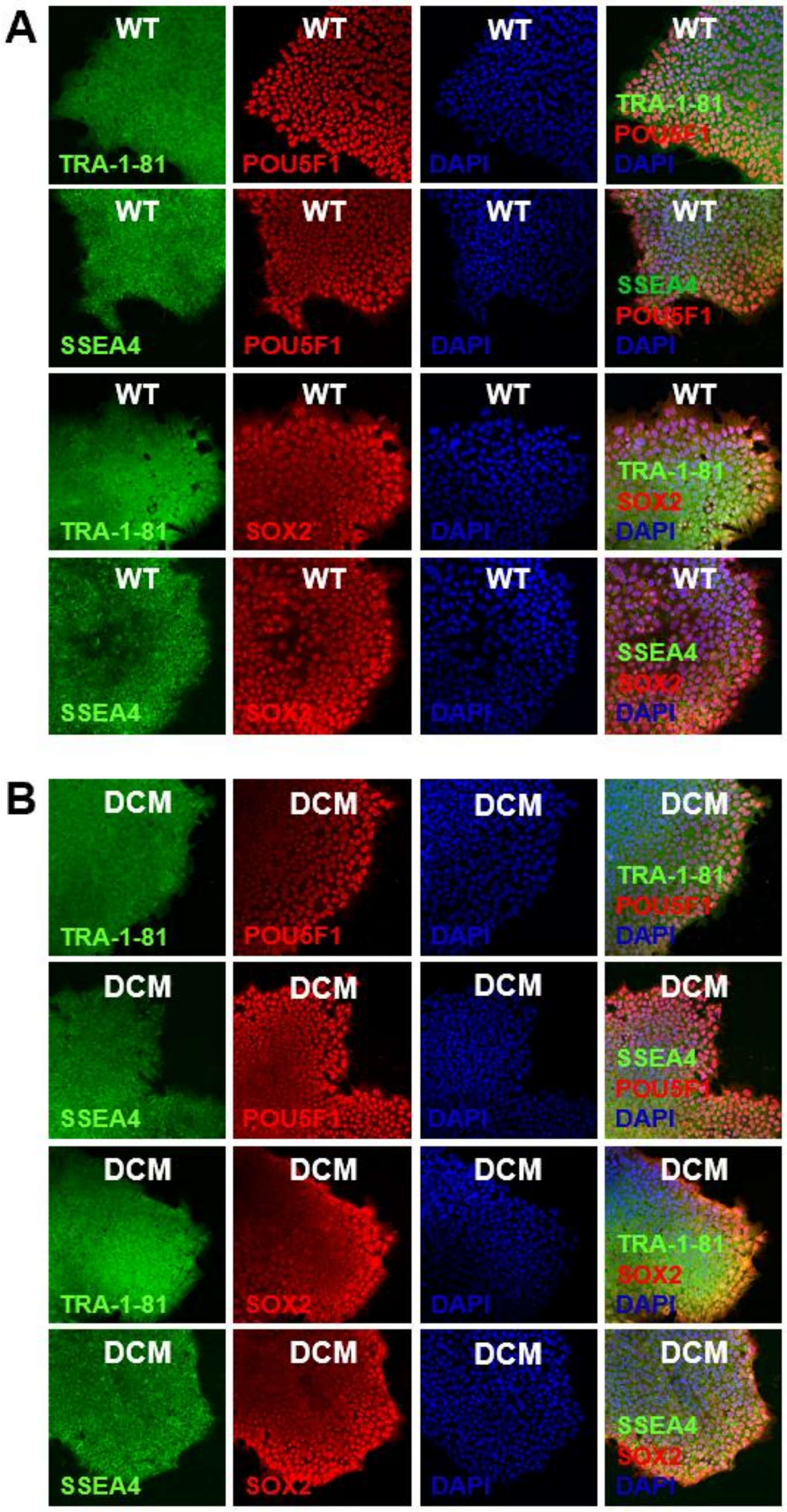

Supplemental Figure 1. Immunostaining for pluripotency markers in WT control and DCM patient-specific iPSCs.

A-B, iPSCs were subjected to immunostaining and confocal imaging for pluripotency markers, tumor-related Antigen-1-81(TRA-1-81), POU class 5 homeobox 1 (POU5F1, Oct 3/4), stage-Specific Embryonic Antigen-4 (SSEA4), and sex determining region Y-box transcription factor 2 (SOX2). **A**, WT control iPSCs; **B**, DCM patient-specific (TPM1-L185F) iPSC. Representative 20x confocal images are shown.

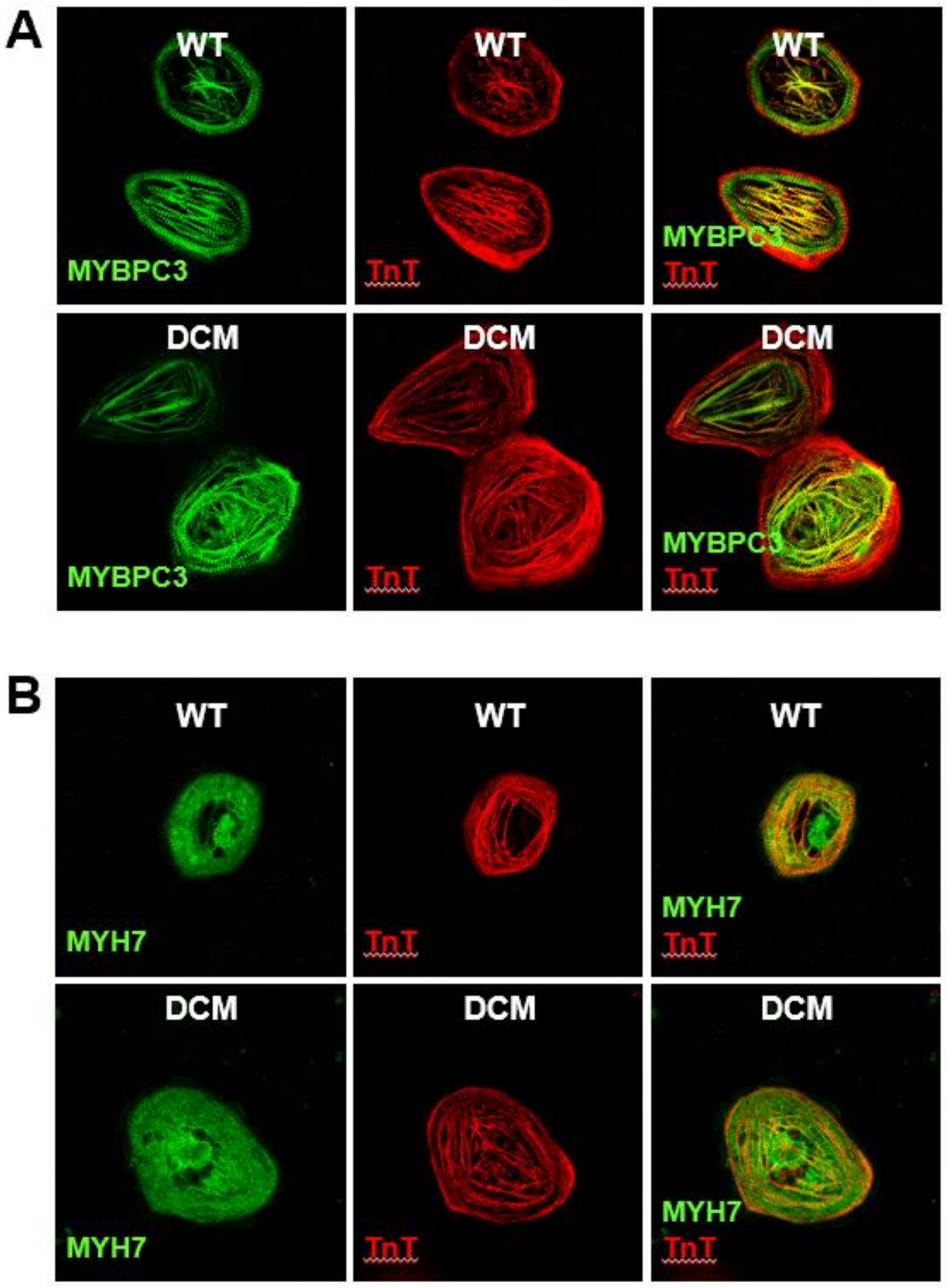

Supplemental Figure 2. Immunostaining for cardiomyocyte markers in WT control and DCM patient-specific iPSC-CMs.

A-B, WT control and DCM patient-specific (TPM1-L185F) iPSC-CMs were subjected to immunostaining and confocal imaging for cardiomyocyte markers. **A**, Myosin binding protein C3 (MYBPC3), troponin T (TnT); **B**, Myosin heavy chain 7 (MYH7), TnT. Representative 63x confocal images are shown.

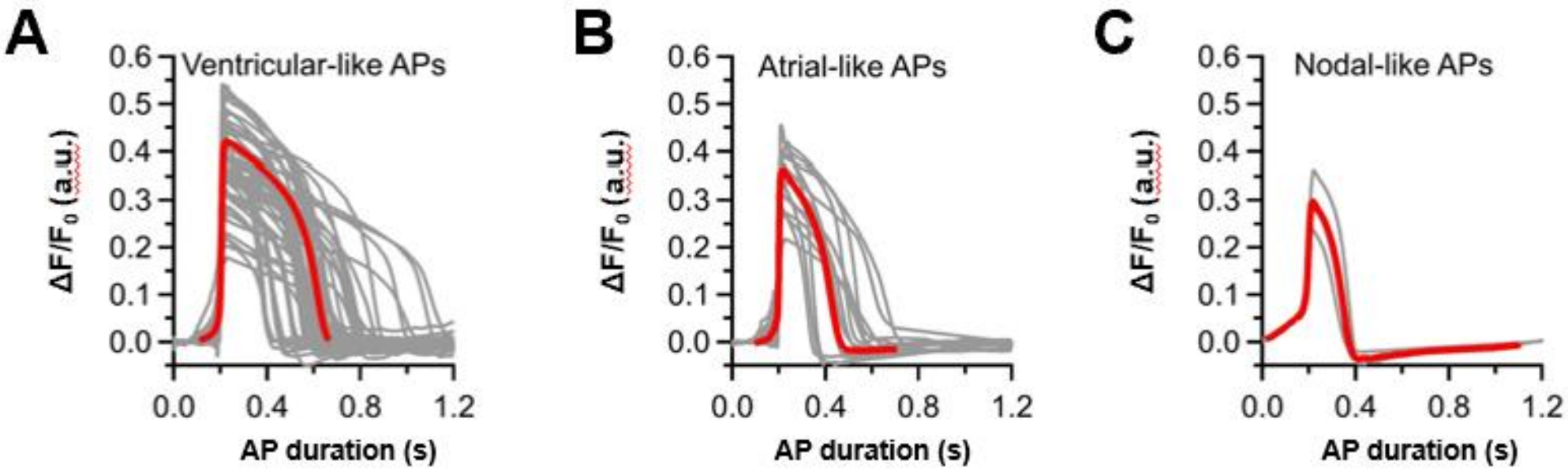

Supplemental Figure 3. Optical action potential mapping.

A-C Optical action potential mapping detected different subtypes of action potentials (APs) iPSC-CMs, such as (**A**) ventricular-like (52 out of 68 cells); (**B**) atrial-like (14 out of 68 cells); or (**C**) nodal-like (2 out of 68 cells).

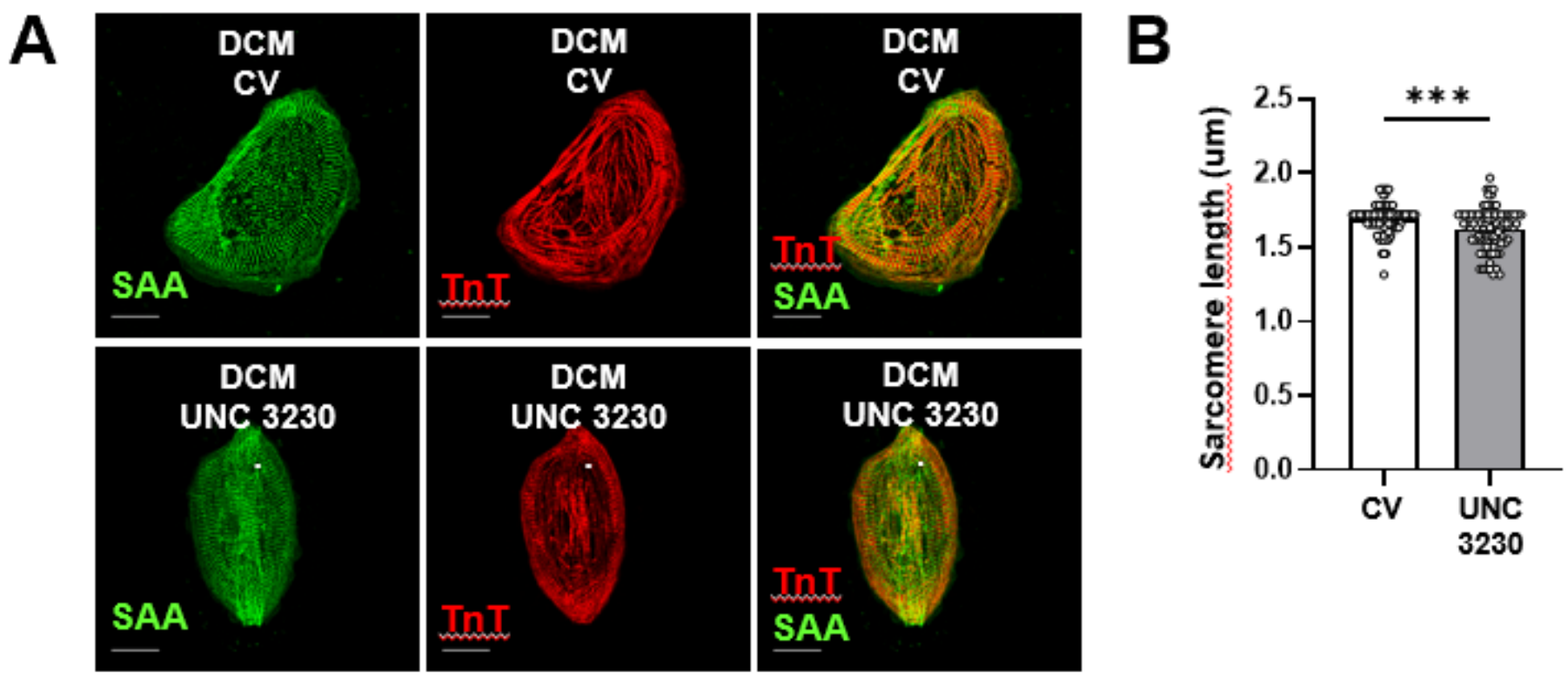

Supplemental Figure 4. Blocking CME-dependent signalling further reduces the sarcomere length of DCM iPSC-CMs.

A-B, PIP5K1C inhibition blocks clathrin-mediated endocytosis (CME)-dependent Fe uptake in DCM iPSC-CMs. DCM iPSC-CMs were treated with control vehicle (CV) or the PIP5K1C-inhibitor UNC3230. **A**, Representative 63x confocal images are shown for cardiac troponin T (TnT, red) and sarcomeric α-actinin (SAA, green); scale bar, 20 µm; **B**, Sarcomere length analysis in DCM iPSC-CMs via the radially averaged magnitude spectrum (RAMS) method; n = 3 independent cardiac differentiations, n = 30 images per experiment; data are shown as mean±SEM. ***P < 0.001 (P < 0.0001; Mann-Whitney U test).

REFERENCES

1. H. Xu *et al.*, Non-negative blind deconvolution for signal processing in a CRISPR-edited iPSC-cardiomyocyte model of dilated cardiomyopathy. *FEBS Lett*, (2021).
2. Y. Dai *et al.*, Troponin destabilization impairs sarcomere-cytoskeleton interactions in iPSC-derived cardiomyocytes from dilated cardiomyopathy patients. *Sci Rep* **10**, 209 (2020).
3. A. V. Malkovskiy *et al.*, Integrated Ca(2+) flux and AFM force analysis in human iPSC-derived cardiomyocytes. *Biol Chem* **402**, 113-121 (2020).
4. D. Silva Dos Santos *et al.*, Empagliflozin reduces arrhythmogenic effects in rat neonatal and human iPSC-derived cardiomyocytes and improves cytosolic calcium handling at least partially independent of NHE1. *Sci Rep* **13**, 8689 (2023).
5. Y. Dai *et al.*, An Alternative Mechanism of Subcellular Iron Uptake Deficiency in Cardiomyocytes. *Circ Res* **133**, e19-e46 (2023).
6. J. Bauersachs, Heart failure drug treatment: the fantastic four. *Eur Heart J* **42**, 681-683 (2021).
7. P. A. Heidenreich *et al.*, 2022 AHA/ACC/HFSA Guideline for the Management of Heart Failure: A Report of the American College of Cardiology/American Heart Association Joint Committee on Clinical Practice Guidelines. *J Am Coll Cardiol* **79**, e263-e421 (2022).
8. M. Writing Committee, A. A. J. C. Members, 2022 AHA/ACC/HFSA Guideline for the Management of Heart Failure. *J Card Fail* **28**, e1-e167 (2022).
9. A. V. Malkovskiy *et al.*, Integrated Ca2+ flux and AFM force analysis in human iPSC-derived cardiomyocytes. *Biol Chem*, (2020).
10. M. Habeck, Saleem, N., Plota, D., Cheruiyot, C., Kohl, T., Lehnart, S., Jakobs, S., Ebert, A., Sarcomere analysis in human cardiomyocytes by computing radial frequency spectra. *bioRxiv*, (2025).
